# DM1 Phenotype Variability and Triplet Repeat Instability: Challenges in the Development of New Therapies

**DOI:** 10.3390/ijms21020457

**Published:** 2020-01-10

**Authors:** Stéphanie Tomé, Geneviève Gourdon

**Affiliations:** Inserm UMR 974, Sorbonne Université, Centre de Recherche en Myologie, Association Institut de Myologie, F-75013 Paris, France; stephanie.tome@inserm.fr

**Keywords:** myotonic dystrophy, clinic variability, CTG repeat instability

## Abstract

Myotonic dystrophy type 1 (DM1) is a complex neuromuscular disease caused by an unstable cytosine thymine guanine (CTG) repeat expansion in the *DMPK* gene. This disease is characterized by high clinical and genetic variability, leading to some difficulties in the diagnosis and prognosis of DM1. Better understanding the origin of this variability is important for developing new challenging therapies and, in particular, for progressing on the path of personalized treatments. Here, we reviewed CTG triplet repeat instability and its modifiers as an important source of phenotypic variability in patients with DM1.

## 1. Introduction

Myotonic dystrophy type 1 (DM1) is a complex disease characterized by multisystemic and variable symptoms [1]. Tremendous progress has been made in recent decades in understanding pathophysiological mechanisms due to expanded CTG repeats, paving the way for new therapeutic developments concomitant with the development of new and powerful therapeutic tools [2]. Preclinical assays for DM1 are underway, exploring all possible approaches, and clinical assays have started or are in the starting blocks [3]. Along with the development of innovative molecular tools to block the disastrous consequences of expanded CTG repeats, the broad clinical spectrum associated with variability in onset and severity of symptoms represents a challenge for patient stratification in order to design future trials.

## 2. DM1: Variable from All Sides

**Figure 1:**
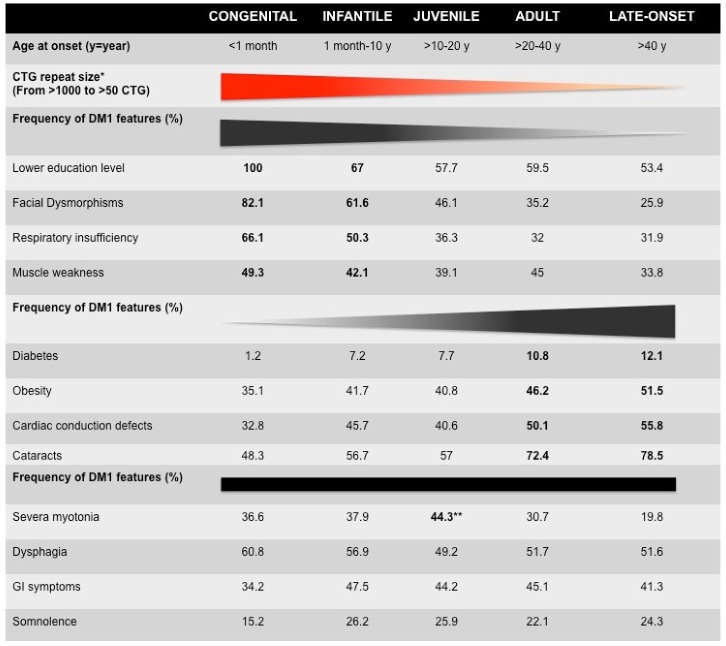
Clinical characteristics of French myotonic dystrophy type 1 (DM1) cohorts [6]. * The mean of CTG repeat length decreases from congenital to late-onset form. The distribution of CTG repeat size overlaps between clinical forms, suggesting genetic variability (see Table 1). The bold numbers represent the highest frequency of certain DM1 features. ** The frequency of most DM1 features among the five forms of DM1 increases or decreases from congenital to late-onset forms. However, the frequency of dysphagia, gastrointestinal (GI) symptoms, and somnolence is stable between the five clinical subtypes. Myotonia is observed in adult form with the highest frequency (72.4%).

From very early descriptions of the disease, DM1 has been recognized as one of the most variable human disorders, with age at onset ranging from fetal to late-adult age and affecting many tissues and systems [1]. Anticipation is particularly evident among DM1 families and has found its molecular basis with the identification of the dynamic CTG repeat expansion [4]. Since then, various laboratories have attempted to correlate, more or less successfully, CTG repeat length, symptoms severity, clinical features, and the classification of clinical forms. The recent developments of registries and cohort studies have made it possible to better characterize the variability of symptoms in populations, and they are valuable tools for correlation studies [5,6,7,8,9,10,11,12,13,14,15]. A recent systematic study performed by the French DM-scope registry has carefully revisited the classification of the disease forms and identified five subtypes in the broad clinical spectrum (Figure 1 and Table 1) [6,7]. These subtypes correlate with the CTG repeat length (CTG size decreased from congenital form (CDM) to late-onset form) but significant overlaps could be observed, highlighting the variability of DM1. This study confirmed the clinical spectrum of DM1 and highlighted the temporal succession of clinical profiles. In addition, sex appears to be an important modifying factor, not only for transmitting the severity of the disease but also for the individual clinical profiles (Table 1) [7].

The main difficulty in diagnosis lies in the CTG repeat length determination, which becomes difficult for long expansions. Polymerase chain reaction (PCR) amplifications are difficult for a high number of CTG repeats, and Southern blotting and triplet-primed (TP)-PCR do not allow accurate CTG number measurements. Although optimized protocols have been developed more recently, precise determination of repeat numbers is limited to ~1000 CTG repeats [16,17]. In addition, age at the time of diagnosis and somatic instability in the blood are confounding factors that can bias correlation studies [18]. Nevertheless, methods have emerged that provide better estimates of the inherited repeat length and extent of the somatic mosaicism in the blood or available tissues. The use of small-pool-PCR (SP-PCR) developed by Monckton et al. in the 1990s has provided considerable information on somatic instability levels in patients and has helped to refine phenotype–genotype correlations in large populations. There is now evidence to clearly demonstrate that length of the estimated inherited repeat length (ePAL) is strongly correlated with age of onset and is the strongest modifier of disease severity [19,20,21]. Persistent somatic instability in various tissues during the lifetimes of DM1 patients also has an important role to play. Although it appears to be strongly correlated with ePAL, the level of somatic instability may vary from one patient to another (even for those with a similar repeat length) and represent a quantitative hereditary trait [19,22]. This trait may be associated with *trans*-acting genetic modifiers that start to emerge, such as the *MutS homolog 3* gene (*MSH3*), and with *cis*-acting factors, such as CTG repeat interruptions (see below).

## 3. Variable CTG Repeats and Mechanisms Involved

### 3.1. Intergenerational Instability

Studies of intergenerational instability in DM1 families have shown that the CTG repeat sequence is highly biased towards expansions in parent–child transmissions, with a low tendency toward contractions. A large pedigree analysis estimated the frequency of contractions at 10% of paternal transmissions and at 3% of maternal transmissions [23]. The dynamic of intergenerational instability depends on the sex and the size of the CTG repeats in the transmitting parents. However, the effect of the sex on the behavior of intergenerational CTG repeats depends on the size of the CTG repeats in affected parents. The expanded CTG < 80 CTG repeat units are unstable, with larger intergenerational expansions during paternal transmissions, consistent with an excess of transmitting grandfathers in DM1 families [24,25,26,27,28,29]. Interestingly, maternal transmissions lead to larger expansions when the affected mother carries a mutated allele from 80 to 250 CTG repeats, consistent with higher CDM cases after maternal transmissions [26]. 

It is difficult to accurately estimate the intergenerational CTG repeat size changes in DM1 families due to the somatic mosaicism observed in DM1 patients in most cases. However, few analyses of CTG repeat size distributions in germ cells, human embryonic stem cells (hESCs), and embryos have shown that intergenerational instability occurs during the first states of gametogenesis and the first postzygotic events [18,30,31,32,33,34,35]. Changes in CTG repeat lengths are already present in spermatozoa and immature oocytes of DM1 patients, suggesting that intergenerational instability occurs mainly during germline cell divisions and/or DNA repairs [30,33]. A DM1 mouse model showed that germinal CTG expansions are already present in spermatogonia, indicating that the mechanism of intergenerational instability is independent of the meiosis process [36]. In human samples, the highest rate of contraction is observed in spermatozoa, with a frequency of 14.3%. No reduction was observed in oocytes [33]. This data is consistent with results showing that intergenerational CTG repeat contractions occur more often during paternal transmissions [23]. Large expansions > 1000 CTG repeats were observed in immature and metaphase II oocytes, whereas these large mutated alleles were absent in the sperm of most DM1 males, consistent with the low frequency of paternal transmissions of CDM cases [33,35]. 

Interestingly, methylation of the sequence located around the expanded repeat might explain, at least in part, the maternal bias for CDM and the transmissions of large expansions. A recent attractive hypothesis has proposed that methylation around the repeats, leading to reduced expression of the *SIX5* gene in the DM1 locus, could be detrimental to spermatogonia and may prevent the transmission of large repeat expansions after paternal transmissions [37]. 

Intergenerational CTG repeat instability is also observed in hESCs isolated from the inner cell mass of mature blastocytes at 6–7 days after fertilization. The hESCs with large repeat expansions (>1500 CTG) show a tendency towards contractions, whereas the hESCs with 250 and 410 CTG repeats show an instability bias towards expansions at the earliest passages [34]. A parental age effect on the CTG intergenerational length changes for men, with fewer than 70 CTG repeat tracts, and women with <250 units were also observed in a large Costa Rican DM1 cohort [26]. The parental age at conception appeared positively correlated with the CTG repeat length changes across transmissions. Although the DM1 mutation was discovered more than 25 years ago and the technology has been improved, the understanding of the behavior of intergenerational CTG repeats remains complex because of the variability among DM1 patients. This variability is associated with the initial size of the repeat, the age and the sex of patients, and the genetic modifiers of the triplet repeat instability. 

### 3.2. Somatic Instability 

CTG repeats become unstable in tissues between 13 and 16 weeks of gestation age, with the largest expansion in heart, skin, and muscles, and continue to expand over time [38,39]. The degree of somatic instability is tissue-specific, repeat length-dependent, and age-dependent. Somatic mosaicism observed in blood is highly biased towards expansions and contributes to the progressive nature of the various symptoms in different DM1 ethnic groups [19,20,21]. The averages of CTG repeat lengths increase over time in DM1 blood samples and depend on the initial sizes of the repeats [20,40,41]. Indeed, the largest repeats show the highest somatic mosaicism [40]. Recently, it was shown that the degree of somatic instability is lower in saliva (noninvasive sampling method) than in blood, with the difference being highest in DM1 patients with >150 CTG repeats. Interestingly, ePAL in blood or saliva only explains 75% and 66% of the variations of the age of onset, respectively, suggesting the role of other modifier factors [42]. Somatic instability may be considered as a beneficial therapeutic target for reducing CTG repeat instability in all affected tissues. Few studies have analyzed the sizes of the repeats between tissues due to reduced accessibility of human tissues. In the 1990s, early data revealed a high degree of heterogeneity in CTG repeat expansions in different tissues by using Southern blotting, with greater CTG repeat lengths in muscles, heart, diaphragm, and testes than blood, cerebellum, spleen, and thymus [30,39,43]. 

In DM1 hESC cells, the data revealed a stabilization of the repeats after arrest of proliferation and differentiation of cells in osteogenic progenitors, neural progenitors, and keratoma cells [34]. Subsequent studies have also shown a stabilization of triplet repeats in induced pluripotent stem cells (iPSCs) and hESC cells after differentiation [44,45]. The data in stem cells evoke a role in proliferation rates in triplet repeat instability in patients. However, no correlation was discovered between the somatic mosaicism observed in tissues and their proliferative rate. In DM1 mouse models, it was shown that CTG repeats can expand rapidly in nondividing cells, further underlying the role of DNA repair in the process of instability [46,47]. In addition, data in DM1 patients and in various models, such as mouse models, or cells have demonstrated that DNA repairs but also DNA replications, transcriptions, and epigenetic changes, as well as the sizes and purity of CTG repeat expansions, influence the dynamics of CTG repeat instability [48,49,50,51,52]. Each process probably participates in a combinatorial manner with relative efficacy that can vary in different tissues and may participate in the variability observed between DM1 patients. 

## 4. DM1 Variability and Modifiers 

The identification of genetic modifiers in DM1 patients is an important step in developing new therapies and particularly in advancing personalized treatments. Over the past decade, triplet-primed PCR (TP-PCR), enzymatic digestion, and classic Sanger sequencing have revealed the existence of repeat interruptions in expanded CTG alleles in DM1 individuals [53,54]. CGG, CCG, CTC, and CAG interruptions account for approximately 4–9% of the DM1 population and are associated with the stabilization of CTG repeat tracts in blood [20,21,51,52,54,55,56,57,58]. Moreover, no intergenerational expansion was observed in parental transmissions, supporting a stabilization effect of interruptions on triplet repeat tracts. Recent data have shown that CCG/CGG interrupted repeats play a key role in the progression of DM1 symptoms and the age of onset, leading to a reduction in the severity of the disease and a delay of age onset [21]. However, the numbers, locations, and types of interruptions were not considered in this study. Conventional technologies do not allow analyses of the sequences in the middle of large CTG repeat expansions, and thus induce a loss of information that can bias the interpretation of current data. Single-molecule real-time sequencing developments by Pacific Biosciences will provide new data to fill the gaps in our knowledge. The characterizations of variants should be strongly considered during routine diagnosis to improve prognosis in the DM1 population and should be used in future clinical trials. 

Several DM1 mouse models have shown the role of the mismatch repair (MMR) pathway in the dynamic of triplet repeat instability, where the MutSß complex (*MSH2–MSH3*) plays a key role in the formation of CTG repeat expansions [59,60,61,62]. In trinucleotide repeat mouse models, *MSH3* expression is an important parameter in the degree of triplet instability and is considered as a limiting factor in this process [59,63,64,65]. Recent data revealed that *MSH3* single-nucleotide polymorphisms are associated with somatic mosaicism rates by potentially modifying *MSH3* expression and/or activity in DM1 large cohorts [66,67]. In addition, genetic association analyses suggest that *MSH3/DHFR* three tandem repeat alleles, named 3a alleles, may reduce somatic rates but also delay the onset in DM1 patients [67]. *MSH3* is clearly a genetic modifier of somatic instability in DM1 but also in other CTG•CAG trinucleotide repeat diseases, suggesting a common mechanism [66,67]. A large analysis of the Huntington Disease (HD) cohort revealed a clear association between several polymorphisms within DNA repair genes, such as *MSH3*, *LIG1*, *FAN1*, and *MLH1*, and the degree of somatic mosaicism, suggesting that *LIG1* or *FAN1* polymorphisms may also explain the variability in DM1 patients, in addition to the MMR proteins [68,69]. 

Epigenetic changes were also suggested as modifiers of triplet repeat instability and phenotypic variability within DM1 patients [22,37]. Pyrosequencing data revealed that DNA methylation within the *DMPK* gene can contribute to phenotypic variability (respiratory parameter and muscle strength), regardless of CTG repeat length [22]. Thus, the epigenome of patients may contribute to the progression of DM1 disorder. However, the environment, including food and air pollution, may affect epigenetic changes and has to be considered in epigenetic analyses [70]. Genomic variants may also affect RNA or proteins, which could explain, in part, the clinical variability observed in DM1 patients. Muscleblind-like splicing regulator 1 (MBNL1) variants have been identified and may be an alternative cause of clinical variability in DM1 [71,72]. The *rs323622* polymorphism in MBNL1 has been associated with a more severe phenotype and may explain about 2% of the variance in disease severity [72]. 

## 5. Concluding Remarks 

Since the discovery of unstable CTG repeats causing DM1, considerable progress has been made in characterizing the dynamics of CTG repeat expansions and their relationship to various aspects of the disease, such as clinical severity, onset, and variability of symptoms. The concomitant development of sophisticated techniques has made it possible to refine the correlation between the size of the repeats and the clinical picture and revealed some of the variability evident in DM1. The identification of genetics and modifiers will progress in the future, and the integration of all parameters should allow for a more accurate prognosis and will facilitate the stratification of patients in future clinical trials.

## Figures and Tables

**Table 1:** Genetic characteristics of French DM1 cohorts [7]. SD = standard deviation.

	Congenital	Infantile	Juvenile	Adult	Late-Onset
CTG repeat size					
Maternal transmission Mean (SD)	1337 (684)	1051 (401)	784 (369)	610 (393)	294 (310)
Paternal transmission Mean (SD)	1190 (711)	760 (376)	668 (399)	538 (359)	346 (340)
